# Creation of Scientific Response Documents for Addressing Product Medical Information Inquiries: Mixed Method Approach Using Artificial Intelligence

**DOI:** 10.2196/55277

**Published:** 2025-03-13

**Authors:** Jerry Lau, Shivani Bisht, Robert Horton, Annamaria Crisan, John Jones, Sandeep Gantotti, Evelyn Hermes-DeSantis

**Affiliations:** 1 phactMI Gainesville, FL United States; 2 Department of Pharmacy Practice and Administration Ernest Mario School of Pharmacy Rutgers, The State University of New Jersey New Brunswick, NJ United States; 3 EMD Serono Boston, MA United States; 4 Eli Lilly and Company Bangalore India; 5 Win-Vector Labs Marin City, CA United States; 6 Pfizer Montreal, QC Canada; 7 Indegene Limited Bangalore India

**Keywords:** AI, LLM, GPT, biopharmaceutical, medical information, content generation, artificial intelligence, pharmaceutical, scientific response, documentation, information, clinical data, strategy, reference, feasibility, development, machine learning, large language model, accuracy, context, traceability, accountability, survey, scientific response documentation, SRD, benefit, content generator, content analysis, Generative Pre-trained Transformer

## Abstract

**Background:**

Pharmaceutical manufacturers address health care professionals’ information needs through scientific response documents (SRDs), offering evidence-based answers to medication and disease state questions. Medical information departments, staffed by medical experts, develop SRDs that provide concise summaries consisting of relevant background information, search strategies, clinical data, and balanced references. With an escalating demand for SRDs and the increasing complexity of therapies, medical information departments are exploring advanced technologies and artificial intelligence (AI) tools like large language models (LLMs) to streamline content development. While AI and LLMs show promise in generating draft responses, a synergistic approach combining an LLM with traditional machine learning classifiers in a series of human-supervised and -curated steps could help address limitations, including hallucinations. This will ensure accuracy, context, traceability, and accountability in the development of the concise clinical data summaries of an SRD.

**Objective:**

This study aims to quantify the challenges of SRD development and develop a framework exploring the feasibility and value addition of integrating AI capabilities in the process of creating concise summaries for an SRD.

**Methods:**

To measure the challenges in SRD development, a survey was conducted by phactMI, a nonprofit consortium of medical information leaders in the pharmaceutical industry, assessing aspects of SRD creation among its member companies. The survey collected data on the time and tediousness of various activities related to SRD development. Another working group, consisting of medical information professionals and data scientists, used AI to aid SRD authoring, focusing on data extraction and abstraction. They used logistic regression on semantic embedding features to train classification models and transformer-based summarization pipelines to generate concise summaries.

**Results:**

Of the 33 companies surveyed, 64% (21/33) opened the survey, and 76% (16/21) of those responded. On average, medical information departments generate 614 new documents and update 1352 documents each year. Respondents considered paraphrasing scientific articles to be the most tedious and time-intensive task. In the project’s second phase, sentence classification models showed the ability to accurately distinguish target categories with receiver operating characteristic scores ranging from 0.67 to 0.85 (all *P*<.001), allowing for accurate data extraction. For data abstraction, the comparison of the bilingual evaluation understudy (BLEU) score and semantic similarity in the paraphrased texts yielded different results among reviewers, with each preferring different trade-offs between these metrics.

**Conclusions:**

This study establishes a framework for integrating LLM and machine learning into SRD development, supported by a pharmaceutical company survey emphasizing the challenges of paraphrasing content. While machine learning models show potential for section identification and content usability assessment in data extraction and abstraction, further optimization and research are essential before full-scale industry implementation. The working group’s insights guide an AI-driven content analysis; address limitations; and advance efficient, precise, and responsive frameworks to assist with pharmaceutical SRD development.

## Introduction

Pharmaceutical manufacturers play a crucial role in meeting health care professionals’ information needs by providing them with scientific response documents (SRDs). These documents provide comprehensive and evidence-based answers to unsolicited questions concerning a medication or disease state [1]. The development and maintenance of SRDs are entrusted to the medical information department within these organizations. This department is composed of medical experts who possess in-depth knowledge of specific therapeutic areas and are responsible for various strategic activities, including the meticulous development of SRDs [2]. SRDs are tailored to address specific inquiries, presenting a concise summary, relevant background information, clinical data, and scientifically balanced references [1]. Considering the escalating demand for SRDs and the increasing complexity of therapies, the role of medical information departments has become more critical than ever. A 2018 survey of 27 pharmaceutical companies revealed that a medical information department creates an average of 716 new SRDs and maintains 2510 existing SRDs annually [2]. Fully developing a new SRD required an average of 31 hours for medical experts, while updating or revising existing SRDs involved an average of 21 hours [2]. Medical information experts use this time to answer the SRD query following a scientific method approach [3]. The strategic and resource-intensive nature of SRD development and the surge in health care professional inquiries emphasize the pressing need for timely and comprehensive information. To address these challenges, there is a growing interest across medical information departments in leveraging advanced technologies and artificial intelligence (AI) tools, such as large language models (LLMs) and traditional machine learning techniques, to enhance and streamline the SRD development process. There are several steps to develop an SRD, including reading articles, selecting article content, paraphrasing article content, creating a citation list, editorial changes, data integrity, and content review. Some of these steps may be more time-consuming than others.

To better understand the current advancements in AI, consider an analogy used in software development. Programming can be thought of as software 1.0, where a machine relies on explicit, step-by-step instructions from a programmer to perform designated tasks. Machine learning represents software 2.0, where developers present labeled examples of input and output data to the machine so that it can identify patterns that allow it to predict outcomes from inputs. This kind of supervised machine learning has enabled rapid progress in many areas of natural language processing, including applications in language translation, sentiment analysis, and information retrieval. More recently, LLMs, such as OpenAI’s Generative Pre-trained Transformer (GPT), are complex machine learning models trained to predict subsequent words in natural language text based on the text so far. This allows the machine to generate statistically plausible output given a “prompt.” Beyond simple prompt completion, such models can be trained to follow instructions in the prompt, such as “Summarize the following paragraph.” Designing prompts that lead an LLM to produce a desired output is a novel and distinct paradigm in software development, which can be classified as “software 3.0” [4].

Language models convert language to numerical representation, and specialized models create semantic embedding by exporting a sentence as a vector of floating-point numbers [5]. By converting concepts into numeric vectors, embeddings enable computers to represent the connections between concepts. The relationship between two embeddings is determined by the vector distance, with smaller distances indicating higher relatedness and larger distances implying lower relatedness. Embeddings are easily consumed and compared by other machine learning models and algorithms for tasks like clustering text strings based on similarity or ranking search results by query relevance. Furthermore, embeddings exhibit semantic similarity—numerically similar embeddings correspond to similar meanings.

**Figure 1:**
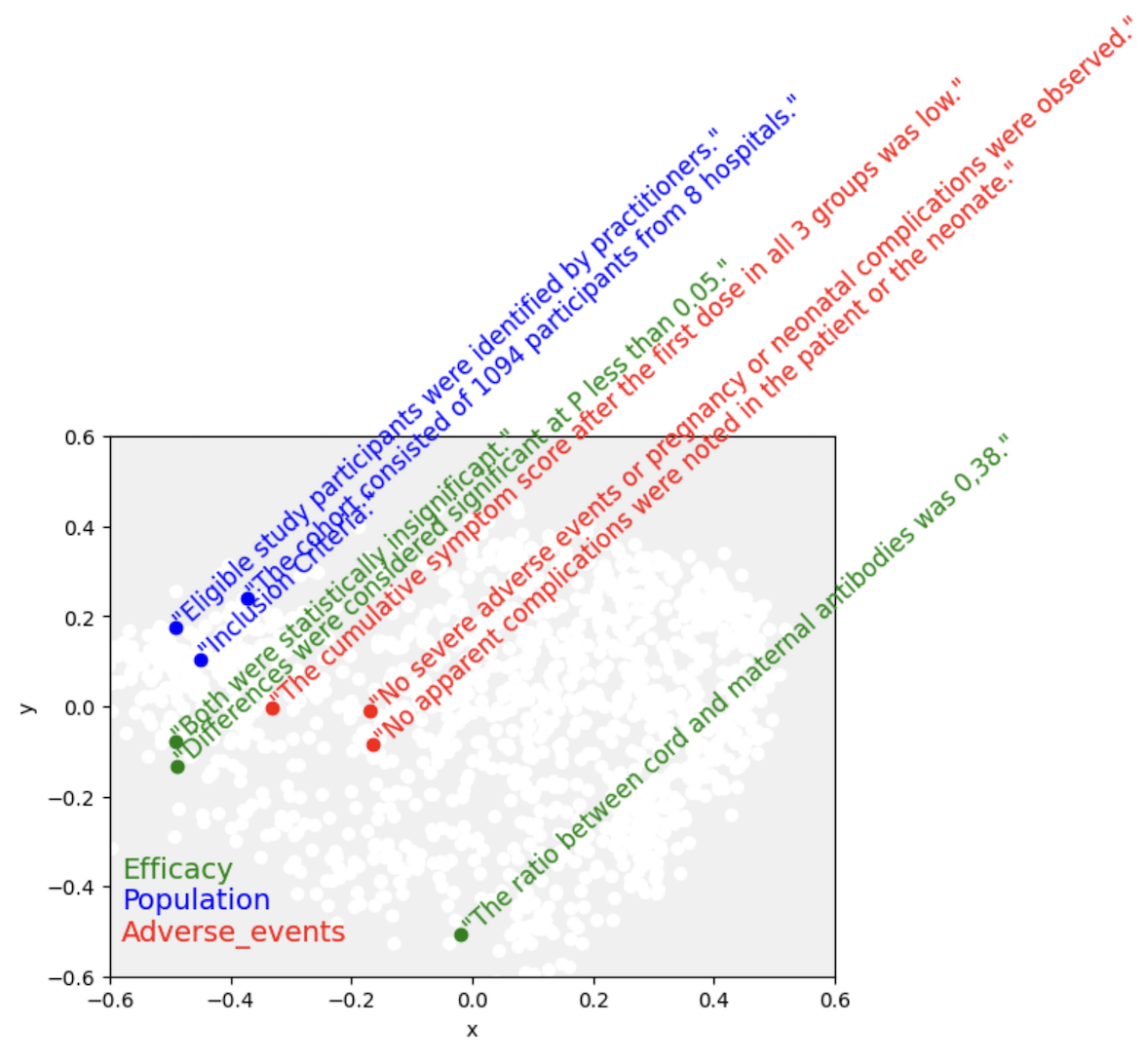
High-dimensional data visualization of embeddings. The t-SNE (t-distributed stochastic neighbor embedding) algorithm was used to transform data into 2 dimensions. Different colors were chosen for different sections based on reviewer feedback (based on the test set used in the study).

Figure 1 shows examples of semantic embeddings of sentences based on the dataset used in this study. The original 768-dimension embeddings were mapped down to 2 dimensions to visualize them, showing that sentences on similar topics are close together. Colors indicate the category to which the sentence belongs. Here, the 3 sentences in blue (“Population”) are close semantically to one another, as are the 3 sentences in red (“Adverse_events”). One of the sentences in the “Efficacy” category is far from the other two, but on examining the sentences, it is considered an outlier talking about a ratio of antibodies, while the two that are close to one another both concern statistical significance.

LLMs apply traditional machine learning concepts and embeddings on a larger scale. Transformers process sequential data, such as natural language, all at once, enabling them to perform tasks like text summarization [6]. GPT is trained to predict the next word using preceding words, capturing linguistic patterns and semantic relationships in large text datasets. GPT often produces coherent and plausible responses. By providing labeled examples, GPT can be fine-tuned for specific tasks to enhance its capabilities. This fine-tuning process allows GPT to adapt its prelearned knowledge to effectively perform tasks such as text generation, question answering, and language translation [7].

AI tools have a well-established history in medicine, with potential applications like artificial neural networks aiding clinical prognosis and diagnosis through pattern recognition first identified in 2004 [8]. Furthermore, within academic and research writing, OpenAI’s ChatGPT has been used to “extract” important information from academic papers (eg, author details, publication date, main findings, etc) and generate summaries of these lengthy papers [9]. However, the use of AI to create medical content, particularly SRDs, is still in its early stages. An April 2023 study showcased the potential of AI by using OpenAI’s ChatGPT to generate draft responses to patient questions based on deidentified information [10]. This pioneering work highlights the need to explore AI’s capabilities in medical content generation in depth.

Although ChatGPT demonstrates impressive language generation abilities, relying solely on it has limitations. ChatGPT, like any LLM, can hallucinate and produce content based on its prediction without logic or fact-checking abilities [11]. Furthermore, there exists a lack of transparency in the training sets used for LLMs like ChatGPT. This, coupled with the complexity of these models, may lead to false or biased information being unintentionally included in the generated content [12]. The accuracy of an SRD is crucial in its creation. Furthermore, traceability and accountability are essential considerations. The use of LLMs like ChatGPT often results in the original authors and sources not being cited, leading to the misattribution of information [13].

This study has 2 aims. The first is to quantify the challenges of SRD creation by gathering the opinions of medical information professionals regarding the time consumption of the various steps of SRD development. To address these challenges and leverage the strengths of both human expertise and AI in the creation of SRDs, a synergistic approach that combines LLM with traditional machine learning classifiers is warranted. The second aim of this study is to develop a framework to explore the feasibility and value addition of integrating AI capabilities, including LLM and machine learning, into the SRD creation process.

## Methods

### Survey of phactMI Members

A working group from phactMI developed a cross-sectional survey to assess the time and tediousness of various aspects of SRD creation. phactMI, a nonprofit consortium of medical information leaders from the pharmaceutical industry, conducted the survey using the survey tool Alchemer. The initial contact for the web-based open survey link was emailed to one contact at each of the 33 member companies in March 2023 (see Multimedia Appendix 1 for email wording). Participation in the survey was voluntary, and no incentives were offered. The survey link was sent once, with one reminder sent during March 2023, and the survey closed on April 15, 2023. The working group pretested the survey using the Alchemer system before distribution. In the recruitment email, the purpose of the survey, length and duration, the lead investigator, and how all data were to be handled were disclosed. Proceeding to the first question was considered consent to participate.

The creation of an SRD is a strategic endeavor comprised of several steps that may be more time-consuming and tedious than others. Specific data collected in the survey included the average time needed for creating an SRD, the average number of papers included in an SRD, etc. Survey respondents were given a list of activities, including paraphrasing article content, creating a citation list, making editorial changes, improving data integrity, selecting article content, reviewing content, and reading articles. Respondents were asked to rank given activities from 1 to 8 in terms of time consumption and tediousness (1 being the most time-consuming or tedious and 8 being the least time-consuming or tedious). The interpretations of time-consuming and tedious were left to the discretion of the survey respondents.

Not all steps had to be ranked by all respondents. A score for each step was created with a weighted calculation, with items ranked first being given a higher value or weight. Weighted values are based on the number of steps selected. The higher the score, the more time-consuming or tedious the steps were considered. The survey results were analyzed to identify those steps in the development of an SRD where the use of AI may offer maximum benefit.

The survey questions were not randomized, and there was no adaptive questioning. There was a total of 10 questions. All questions were displayed on the same page, so no back button or review step was necessary.

Only 1 response per company was allowed. Data were analyzed using descriptive statistics. The full survey questionnaire is provided in Multimedia Appendix 2. The Checklist for Reporting Results of Internet E-Surveys (CHERRIES) for this survey is provided in Multimedia Appendix 3.

### Ethical Considerations

The survey was not approved by an institutional review board as it was not considered human subject data. All survey data were deidentified, saved, and reported in aggregate.

### Authoring SRD

**Figure 2:**
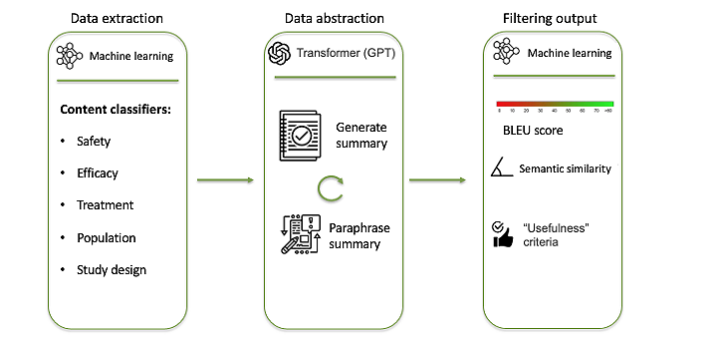
Proposed process design. BLEU: bilingual evaluation understudy; GPT: Generative Pre-trained Transformer.

Another working group consisting of medical information professionals and data scientists was created. Their goal was to leverage AI to support the medical information department’s creation of SRDs. Their aim was to develop a tool that could process scientific articles (input) and provide concise summaries (output). The group identified two key steps in the document authoring process: data extraction and data abstraction. Their problem was figuring out the process between the input and output (Figure 2). Data extraction is the selection of key sentences from publications that address all the data points authors would want to include in a response document, and data abstraction is the generation of a summary of extracted data, followed by paraphrasing to avoid plagiarism of original texts.

### Data Extraction and Machine Training

The working group selected scientific texts from the PubMed Central database focusing on clinical drug trials for data extraction. The narrative text from these articles, excluding text in tables, was extracted, cleaned, and placed into Prodigy, a data annotation tool. A total of 3 domain experts and medical information specialists labeled sentences from narrative text into 5 classifications: safety, efficacy, treatment, population, and study design. These classifications correspond to the main sections of a clinical trial used in the creation of an SRD. A fourth domain expert, a data editor, reviewed all the labels to ensure the labeling criteria were applied consistently. These labeled data were then fed into logistic regression classification models to train the models on identification. The training dataset is available in Multimedia Appendix 4.

Participating companies provided 3 SRDs to the working group. The team extracted clean, narrative text from the provided documents to feed into the models. The models categorized each sentence based on their previous training. Reviewers evaluated and assessed model classifications. Trained models’ performance was evaluated with a receiver operating characteristic (ROC) curve plotting the true positive rate (TPR) and false positive rate (FPR). The area under the curve (AUC) provides an aggregate measure of performance across all possible thresholds, with a higher AUC indicating better performance of the model. A Wilcoxon-Mann-Whitney *U* test statistic was applied.

### Data Abstraction

Summarizing the extracted data was the initial step in data abstraction. The working group used the Hugging Face transformers summarization pipeline leveraging the Facebook/BART-large-cnn model, a language model trained for summarization. The second step was to rewrite and synthesize the extracted text without plagiarizing the original reference by using the GPT-3 model (text-davinci-003). The model received the prompt “Paraphrase this without plagiarizing,” followed by the summarized text. Multiple paraphrases were generated for each input.

### Filtering Output

A total of 2 criteria were used to sort and rank the paraphrased texts: semantic similarities and bilingual evaluation understudy (BLEU) scores. Semantic similarity, measured using cosine similarity between sentence transformer embeddings (distiluse-base-multilingual-cased-v2), assessed the likeness in meaning between the paraphrased sentences and the original text. The greater the semantic similarity between the two sentences, the better the quality of the paraphrasing. The second criterion was the BLEU score, which measured the similarity in word or phrase use between a generated text and the original text. It was calculated using *sacrebleu* with *effective_order* set to true. A low BLEU score reflects a higher quality of paraphrasing, as it indicates less similarity in words and phrases with the original text. Finding the right balance between semantic and textual similarities was crucial for the overall paraphrasing quality. Human reviewers then evaluated the paraphrased text and ranked the text by usefulness with rationales provided.

Throughout the study, the working group fostered collaboration between medical information professionals and data scientists to validate the results. Results from each step were edited by hand to make sure that the next step had clean inputs.

## Results

### Survey of phactMI Members

A total of 21 of the 33 pharmaceutical member companies, based on IP address, opened the survey (view rate 64%). A total of 16 pharmaceutical member companies participated in the survey (participation rate 76%, 16/21), with a completion rate of 81% (13/16). No cookies were used to assign user identification. Duplicate entries were identified by either IP address or company name (if provided). The most complete or most recent entry was kept for analysis. All data from unique entries were included in the analysis.

**Figure 3:**
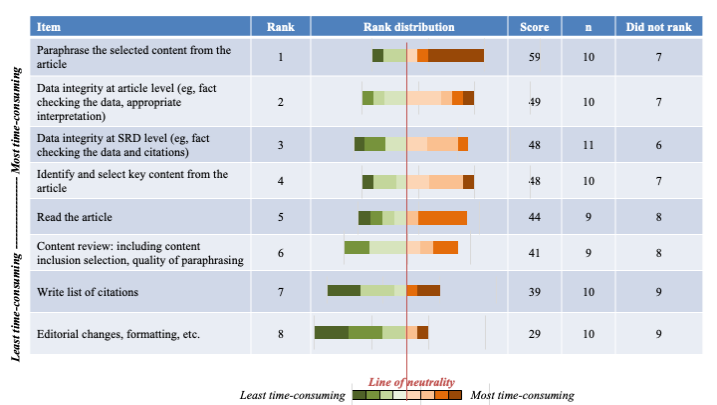
Ranking of steps deemed time-consuming by survey respondents. SRD: scientific response document.

**Figure 4:**
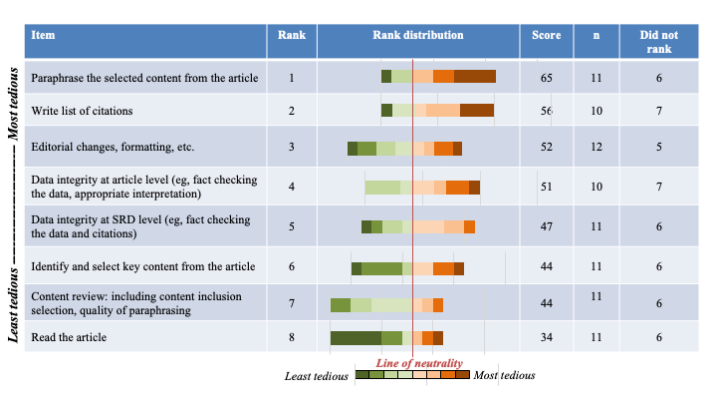
Rankings of tasks deemed tedious by survey respondents. SRD: scientific response document.

On average, a medical information department creates 614 (range: low to 2676) new SRDs and updates or revises 1352 (range: low to 6057) SRDs annually. Respondents indicate it takes, on average, 8.3 hours to create a new SRD and 3.8 hours to update an SRD. In addition, 87% (14/16) of respondents included content from at least 4 studies in SRDs summarizing clinical trial data. The survey results revealed that the top 3 most time-consuming steps in SRD development were paraphrasing study content, checking the data integrity of the paraphrased text versus the source publications, and checking the data integrity of the SRD (eg, checking that the text is cited to the correct publications; Figure 3). While paraphrasing article content was also the most tedious step, the other top steps differed, with writing citations and editorial changes rounding out the top 3 (Figure 4).

### Data Extraction

**Figure 5:**
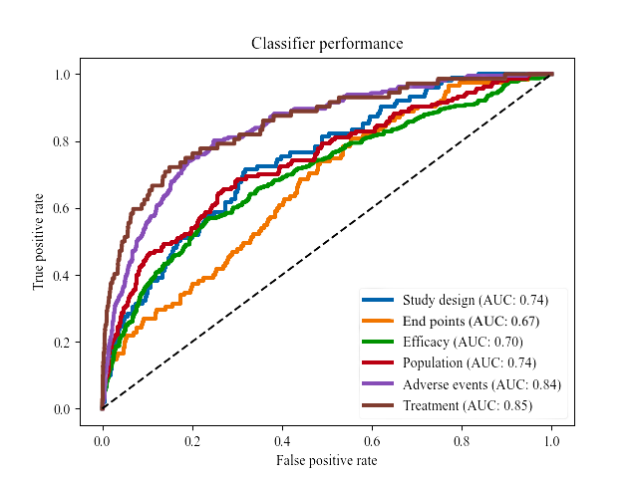
Receiver operating characteristic (ROC) curve of machine learning models’ performance. AUC: area under the curve.

ROC curves are a fundamental way to evaluate classifier performance. AUC values can range from 0.5 to 1.0, with values closer to 1.0 indicating that the classifier’s performance is better than random. Using 3187 sentence data points, ROC curves were generated to assess the classifier’s performance (Figure 5). The model trained on treatment data had the highest AUC (0.85). Models trained on Prodigy data achieved AUC scores of 0.84, 0.74, 0.7, 0.67, and 0.74 for adverse events, population, efficacy, end points, and study design, respectively. The AUC scores for all classifier models exceeded 0.5 (random chance) with *P*<.001. Overall, the model was able to classify sentences appropriately and be better than a random chance (indicated by the dashed line).

### Data Abstraction and Filtering Output

The clinical trial data were inputted into the LLM model to produce an AI-generated summary and an AI-generated paraphrase of the text. Table 1 provides 2 examples of the paraphrasing process, showing the input in the LLM model and output using the prompt “Paraphrase this without plagiarizing.”

In Table 1, the colors correlate to the BLEU scores, with the reddest color being 100 and the bluest color being 0 (as shown by the scale on the right). The y-axis corresponds to the original sentences in the text (0, 1, 2, etc), and the x-axis corresponds to the paraphrased sentences (0, 1, and 2). Each original sentence is compared with each paraphrased sentence with a BLEU score. The lower the BLEU score, the fewer words and phrases there are in common between the original and the paraphrased sentences. Semantic similarity is the overall similarity between the two texts.

**Table 1:** Artificial intelligence (AI)–generated summarized and paraphrased text examples.

Original text input	AI-generated summarized text:	AI-generated paraphrased text:
In the sitagliptin group and glimepiride group, serious adverse experiences were reported for 16 and 11 patients, respectively. One serious adverse experience that was considered drug-related by the investigator was reported in the study: a 61-year-old female in the sitagliptin group experienced a serious drug-related adverse experience of thrombocytopenia (platelet count of 12 000/μL) resulting in discontinuation from the study. Apart from hypoglycemia, the only other adverse event with an incidence of at least 5% in either treatment group was nasopharyngitis (4.8 and 6.9% in the sitagliptin and glimepiride groups, respectively). Overall, clinical adverse experiences and drug-related adverse experiences were reported more frequently with glimepiride, primarily as a result of an increased incidence of hypoglycemia.	In the sitagliptin group and glimepiride group, serious adverse experiences were reported for 16 and 11 patients, respectively. The only other adverse event with an incidence of at least 5% in either treatment group was nasopharyngitis (4.8 and 6.9%).	In the sitagliptin group, 16 patients reported a serious adverse experience. In the glimepiride group, 11 patients reported a serious adverse experience. The only other adverse event with an incidence of at least 5% was nasopharyngitis. 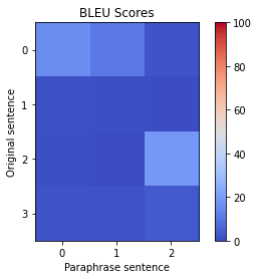 Semantic similarity: 0.76 max BLEU^a^: 18.54
Specific clinical adverse experiences of interest included hypoglycemia and prespecified selected gastrointestinal adverse experiences (abdominal pain, nausea, vomiting, and diarrhea). The incidences of serious clinical adverse experiences were similar between treatment groups. For serious adverse experiences that were considered by the investigator to be related to the study drug, there were three in the glipizide group (myocardial infarction, spontaneous abortion, and hydronephrosis) and one in the sitagliptin group (thrombocytopenia). The overall incidence of infection-related adverse experiences was similar in the two treatment groups. Of the adverse experiences with a higher incidence in the glipizide group, the 95% CI around the between-group difference in incidence excluded zero for cataracts, toothache, hypoglycemia, and hypoesthesia (Table 5). With the exception of hypoglycemia, these adverse experiences (occurring in either group) were generally rated as mild in intensity, not considered related to the study drug, and resolved while patients continued on the study drug. AE, adverse experience. Overall, clinical adverse experiences and drug-related adverse experiences were observed more often with glipizide, largely related to an increased incidence of hypoglycemia. The number of serious adverse experiences was similar in both treatment groups, and no specific pattern of serious adverse experiences was observed in either treatment group.	The incidences of serious clinical adverse experiences were similar between treatment groups. Serious adverse experiences that were considered by the investigator to be related to the study drug were three in the glipizide group (myocardial infarction, spontaneous abortion, and hydronephrosis) and one in the sitagliptin group (thrombocytopenia). The overall incidence of infection-related adverse experiences was similar.	There were similar incidences of serious clinical adverse experiences between treatment groups. The serious adverse experiences that were considered by the investigator to be related to the study drug were three in the glipizide group (myocardial infarction, spontaneous abortion, and hydronephrosis) and one in the sitagliptin group (thrombocytopenia). The overall incidence of infection-related adverse experiences was similar. 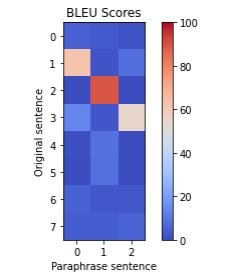 Semantic similarity: 0.86 max BLEU: 89.34

^a^BLEU: bilingual evaluation understudy.

**Figure 6:**
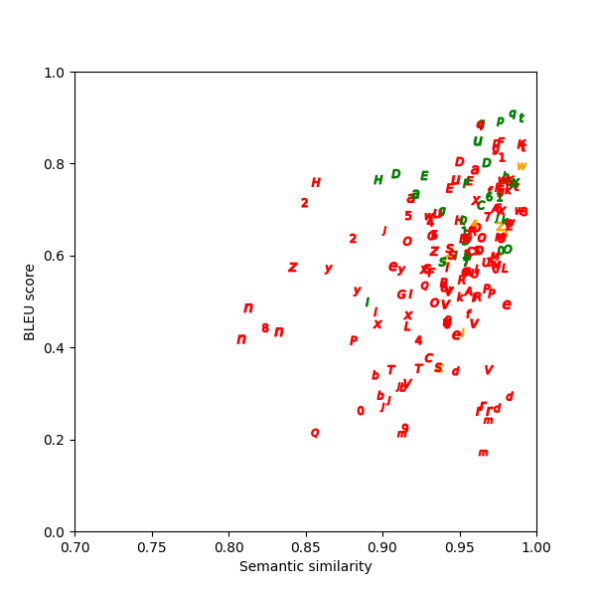
Bilingual evaluation understudy (BLEU) score versus semantic similarity for user 1.

**Figure 7:**
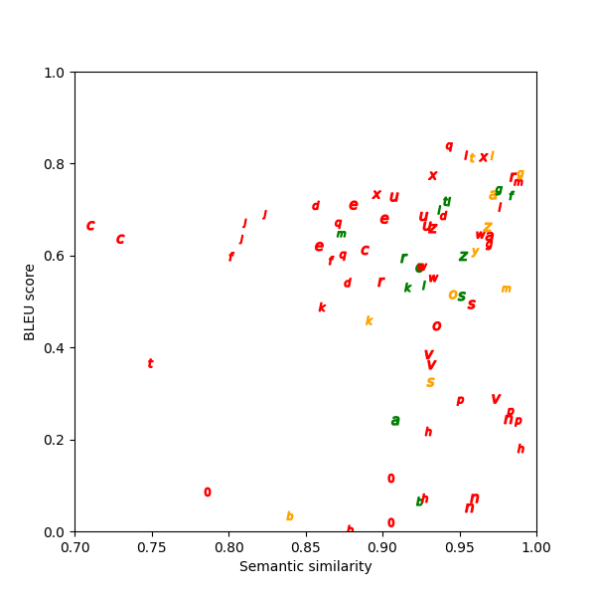
Bilingual evaluation understudy (BLEU) score versus semantic similarity for user 2.

**Figure 8:**
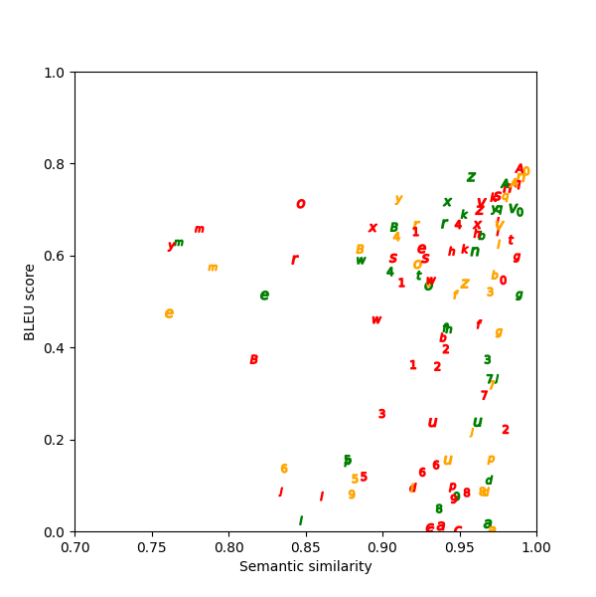
Bilingual evaluation understudy (BLEU) score versus semantic similarity for user 3.

Semantic similarity and BLEU scores calculated for the paraphrases show that in the two example scenarios in Table 1, one has a low BLEU score, and the other has a high BLEU score. The high BLEU score in example 2 contains more original text in the paraphrase compared to the low BLEU score in example 1. Figures 6-8 depict the graphed comparison of BLEU score and semantic similarity among three reviewers (users) to define usefulness. User 2 showed a preference for high semantic similarity, but there was no clear trend with the BLEU score. User 3 consistently favored paraphrases with both high semantic similarity and BLEU score. User 1 had no clear preference trend. Differences between what human reviewers found useful in paraphrases were noted.

## Discussion

### Principal Findings

In the survey section of this study, we found that in the strategic activity of creating SRDs, the major challenge was in paraphrasing articles. In the subsequent phase of this study, traditional machine learning classifiers and LLMs automated portions of the clinical trial summarization process of creating an SRD.

Our survey revealed a much shorter time (8.7 hours and 3.8 hours) to create or revise an SRD compared with the 2018 phactMI benchmarking survey (31 hours and 21 hours) [2]. The variations and limited external validity of the overall survey may be attributed to the nature of the survey, the number of responses, and survey types. Nevertheless, the survey’s results continue to be valuable, as they offer nuanced insights from engaged participants and contribute to our understanding. Regardless of the amount of time, providing solutions to improve the efficiency of creating an SRD would be welcomed.

### Data Extraction

Our study reveals the promise of machine learning models in classifying individual sections within scientific documents, particularly in the context of addressing inquiries within the pharmaceutical industry. The results from the ROC curves suggest that our classifier models outperform random guessing, demonstrating the highest AUC values for the treatment and adverse events classifiers. The transparency and interpretability of our classifier models were pivotal strengths. Unlike LLMs, which are known for their opaqueness in decision-making, our traditional machine learning models have successfully identified and resolved training logic deviation issues. Having clear explanations in the output is invaluable for trust, accountability, and enhancing the models.

In addition, the classifier models exhibited resource efficiency. While finetuning LLMs is a resource-intensive process, we found that adjusting the logistic regression model can be executed in seconds. This efficiency has major implications for rapid model development and deployment.

Enhancing classifier performance necessitates the consideration of several key factors that the working group identified. These factors include providing additional context, predefining known key terminology for specific sections, and exploring methods to reduce false negatives. In future iterations, our approach will expand the scope of classifiers beyond section identification to assess the usefulness of the identified content for inclusion in an SRD. This transition marks a shift from mere classification to a more profound evaluation of content, offering applications in content retrieval tailored to individual user needs.

### Data Abstraction and Filtering Output

Our exploration of paraphrasing performance in LLMs has been highly informative. Quantitative assessment of paraphrased content requires robust tools like semantic similarity and BLEU scores. By leveraging these tools, we gain a deeper understanding of the effectiveness of paraphrasing, ensuring that content retains its intended meaning while being substantially different from the original in terms of phrasing or wording.

The observed variability in LLM-generated paraphrases highlights the difficulty of consistently fine-tuning an LLM for paraphrasing. The diverse approaches to paraphrasing are highlighted by the distinct preferences of human reviewers. Developing a universal model for all preferences is an ambitious endeavor. The working group proposed an alternative approach to this challenge: using simple models that offer users multiple paraphrase options. We can enhance the content ranking and establish core data by providing choices and using smaller datasets, as user selections can potentially be used to train classifiers to identify the kind of content that the user prefers.

The working group also recommends the following next steps with LLMs to further this exercise: (1) fine-tuning an LLM for medical text, (2) better prompt engineering, and (3) LLMs with better citation training. Incorporating these considerations into our discussion of paraphrasing performance and prospects, we navigate the evolving landscape of AI-driven content generation in the pharmaceutical industry. These insights not only promise enhanced content but also embody a user-centric approach that empowers industry professionals to access tailored, high-quality content.

### Need for Human Control in AI-Assisted Scientific Writing

A recent study used ChatGPT to obtain medical information and treatment options for shoulder impingement syndrome [14]. While ChatGPT’s answers were useful for patients, it sometimes provided inaccurate information (prevalence reported with no evidence supporting the number) and biased information (risk factors reported that are not established). Goodman et al [15] conducted a cross-sectional study corroborating these limitations of LLMs. Most responses were accurate and comprehensive, indicating the potential use of LLMs. Occasionally, incorrect answers were provided, and the chatbot provided inaccurate citations when asked for the source of information. Other studies have demonstrated similar drawbacks (misinterpretation of medical terms, hallucination, missed information, factually incorrect statements, and fabricated references) in the use of LLMs in scientific writing and simplified radiology reports [13,15,16]. Accuracy, lack of bias, and traceability to the original publication are crucial in medical information. Thus, using LLMs without considerable human intervention for medical information responses or SRDs is a highly risky proposition. While AI can help humans create a “first draft” of the final SRD, it is imperative for the human writer to retain control over the tool’s input, data extraction for the SRD, and the ultimate inclusion of paraphrased content in the SRD. Our approach includes various “checkpoints” during AI-assisted SRD creation, allowing human writers to intervene and enhance the content’s credibility.

The use of LLMs for scientific writing also presents concerns regarding plagiarism and the use of nonacademic language [13,17]. In addition, LLMs are unable to determine the credibility of their information sources, for example, a blog post versus a PubMed-indexed paper [15]. Our model can overcome numerous limitations by integrating machine learning and LLM systems.

### Limitations

Despite the working group’s diligent effort to maintain scientific rigor in this study, several limitations warrant consideration. The classical machine learning classifiers may have biased models due to training on a constrained dataset and limited reviewer assessments. Instead of relying on experts to label more examples, it may be more efficient to extract labeled examples from existing datasets (eg, adverse events sections from full-text papers in PubMed Central). The use of LLMs like GPT presented known challenges for paraphrasing medical text, such as generative AI issues of “hallucination,” lack of transparency, bias, and privacy concerns [18].

The dynamic generative AI landscape implies that the findings of paraphrase exercises only reflect a snapshot in time. OpenAI introduced GPT-4 Turbo, a 2023 model trained on a larger dataset, while we were drafting this manuscript [13]. Nori et al [19] demonstrated that prompt engineering with GPT-4 outperformed fine-tuned medical models for question answering. The framework described in this paper is similar to the emerging pattern of retrieval augmented generation [20] in leveraging LLMs. The focus of retrieval augmented generation is to provide the LLM with accurate, up-to-date information [20]. The same business drivers from the medical information space prompted this evolution, driven by a need for accuracy and content lineage tracking. The fact that several others have reached a similar conclusion on integrating LLMs into highly regulated industries such as drug manufacturing is a strong validation.

### Conclusions

This study sought to identify the challenges inherent in the development of SRDs and to establish a framework for integrating LLM and machine learning into the SRD creation process. Our tool leverages LLMs and machine learning to enhance AI applications in the pharmaceutical realm. Integrating these two technologies not only saves resources but also addresses major challenges associated with LLMs. Our models can clearly identify sections, paraphrase effectively, and assess content usefulness. These initial findings suggest that machine learning classifiers can predict, to some extent, the sentences authors will choose for summarization and paraphrases they will find useful. Even a modest ability to rank results could improve the suggestions’ quality beyond random. However, the current tool does not have the capacity to generate an SRD for the pharmaceutical sector using zero-shot classification. Nevertheless, it underscores the essential role of traditional machine learning in enhancing future AI models, moving us closer to efficient content handling in the industry. This model has the potential to be a valuable tool in the medical information domain of the pharmaceutical industry, augmenting the efficiency of human document creators, thereby optimizing workflows and improving the quality of services. Further research is required for the optimization, refinement, and validation of these models, using larger training sets and multiple reviewers, before full-scale implementation in the industry.

## Data Availability

All data generated or analyzed during this study are included in this published article and its supplementary information files.

## Appendix

Multimedia Appendix 1 Email invitation for survey participation.

Multimedia Appendix 2 Survey questions.

Multimedia Appendix 3 Checklist for Reporting Results of Internet E-Survey (CHERRIES).

Multimedia Appendix 4 Training dataset.

## Abbreviations

AE: adverse experience
AI: artificial intelligence
AUC: area under the curve
BLEU: bilingual evaluation understudy
CHERRIES: Checklist for Reporting Results of Internet E-Surveys
FPR: false positive rate
GPT: Generative Pre-trained Transformer
LLM: large language model
ROC: receiver operating characteristic
SRD: scientific response document
TPR: true positive rate
