# Supplementary material for: Creation of Scientific Response Documents for Addressing Product Medical Information Inquiries: Mixed Method Approach Using Artificial Intelligence
Source: JMIR AI. 2025 Mar 13;4:e55277. doi: 10.2196/55277 (PMC11950693; doi:10.2196/55277)
Supplement: Multimedia Appendix 1 [file ai_v4i1e55277_app1.docx]

**Appendix: Email for survey distribution**

Dear phactMI Survey Team,
Thank you for your time today, your responses mean a lot to the phactMI members asking these questions. In total there are a maximum of 10 questions and it should take less than 5 minutes to complete. Please have information concerning number of response documents created/reviewed and approximate times. 

Time and effort in creating SRDs from clinical trial data - 10 questions
This data will be used by the AI Content generation team to help understand where AI technology can best be utilize to assist in the SRD process. While we understand that specific data may be difficult to provide since time and effort depend on each individual SRD, we are asking for general and estimate numbers based on summarizing clinical trial data. 

All responses will be kept confidential and only de-identified data will be provided to the survey initiator. Please note that all de-identified survey results will shared with phactMI members via the member portal, and may be used in presentations and publications. If you have any questions about this, previous surveys, or potential future surveys, please reach out to Evelyn at [Evelyn@phactMI.org](mailto:Evelyn@phactMI.org)

By continuing with the survey, you are agreeing to participate.

Evelyn DeSantis
