# Supplementary material for: Creation of Scientific Response Documents for Addressing Product Medical Information Inquiries: Mixed Method Approach Using Artificial Intelligence
Source: JMIR AI. 2025 Mar 13;4:e55277. doi: 10.2196/55277 (PMC11950693; doi:10.2196/55277)
Supplement: Multimedia Appendix 2 [file ai_v4i1e55277_app2.docx]

Time and effort in creating SRDs from clinical trial data – Survey

For the next set of questions., Please provide a single estimate or average. We understand that most of the answers will vary depending on many factors. We are trying to get an overall sense of time and effort.

1. What is the number of scientific response documents created in your MI department in 2022 (Jan - Dec)?
2. What is the number of scientific response documents that are updated in your MI department In 2022 (Jan - Dec)?
3. On average, how long does it take to create a new scientific response document summarizing clinical trial data in your department (In hours)?
4. On average, how long does it take to revise/ update a scientific response document summarizing clinical trial data in your department (in hours)?
5. What is the average number of articles that are included in your typical scientific response document that summarizes clinical trial data?
   1. 1
   2. 2-3
   3. 4-5
   4. 6-7
   5. More than 7
6. How often do you extract data from a table and incorporate it into text in your scientific response documents summarizing clinical trial data?
   1. Always (every SRD)
   2. Frequently (~75% of the SRDs)
   3. Sometimes (~50% of the SRDs)
   4. Rarely (~25% of the SRDs)
   5. Never
7. Rate your agreement with this statement. Data extraction from a table is tedious and time consuming.
   1. Strongly agree
   2. Agree
   3. Neutral
   4. Disagree
   5. Strongly disagree
8. On average, please provide an estimate (in minutes) to summarize one phase three clinical trial article in a scientific response document.
   1. Step 1. Read the article.
   2. Step 2. Identify/select key content from the article.
   3. Step 3. Paraphrase this selected content from Step 2.
   4. Step 4. Data integrity checking.
9. Please rank the following steps in the creation of an SRD summarizing clinical trial data in terms of being time consuming (i.e., Length of time to complete task) with 1being the most time consuming and 8 being the least time consuming.
   1. Read the article.
   2. Identify/select key content from the article.
   3. Paraphrase the select content from the article.
   4. Data integrity at the article level. (e.g. fact checking the data, appropriate interpretation)
   5. Write list of citations.
   6. Content review: Including content inclusion selection, quality of paraphrasing.
   7. Data integrity at SRD level (e.g. fact checking the data and citations)
   8. Editorial changes, formatting, etc.
10. Please rank the following steps in the creation of your SRD summarizing clinical trial data in terms of being tedious, with 1 being the most tedious and 8 being the least tedious.
    1. Read the article.
    2. Identify/select key content from the article.
    3. Paraphrase the select content from the article.
    4. Data integrity at the article level. (e.g. fact checking the data, appropriate interpretation)
    5. Write list of citations.
    6. Content review: Including content inclusion selection, quality of paraphrasing.
    7. Data integrity at SRD level (e.g. fact checking the data and citations)
    8. Editorial changes, formatting, etc.
